# Non-nutritive sweeteners and body weight in children with type 1 and type 2 diabetes: a critical narrative review of current evidence, methodological challenges, biological mechanisms, and clinical implications

**DOI:** 10.3389/fnut.2026.1892085

**Published:** 2026-08-27

**Authors:** Sousana K. Papadopoulou, Exakousti-Petroula Angelakou, Theodosios Koimtsidis, Eleni Pavlidou, Dimitrios Tasoulas, Constantinos Giaginis

**Affiliations:** 1 Department of Nutritional Sciences and Dietetics, School of Health Sciences, International Hellenic University, Thessaloniki, Greece; 2 Department of Food Science and Nutrition, School of Environment, University of Aegean, Lemnos, Greece; 3 Second Department of Cardiology, General Hospital of Nikea-Piraeus, Athens, Greece

**Keywords:** appetite regulation, artificial sweeteners, body weight regulation, childhood obesity, dietary management, glycaemic control, non-nutritive sweeteners, pediatric diabetes

## Abstract

**Introduction:**

Body weight management is an important component of pediatric diabetes care, although its clinical relevance differs between type 1 diabetes (T1D) and type 2 diabetes (T2D) because of distinct pathophysiology, insulin physiology, and therapeutic goals. Non-nutritive sweeteners (NNSs) are widely used to replace added sugars, reducing glycaemic load and, in some settings, energy intake. However, their long-term effects on body weight in children with diabetes remain controversial. This narrative review evaluates current evidence on the association between NNS consumption and body weight in children with T1D and T2D, summarizes proposed biological mechanisms, and discusses methodological and clinical implications.

**Methods:**

A comprehensive narrative literature search identified studies published through April 2026. Randomized controlled trials (RCTs), observational studies, systematic reviews, and meta-analyses evaluating NNS consumption and body weight in pediatric populations were included. Given the limited diabetes-specific evidence, relevant mechanistic findings from adult, animal, and *in vitro* studies were also considered. Evidence was synthesized according to study design, diabetes type, biological mechanisms, methodological limitations, and clinical relevance.

**Results:**

RCTs, conducted mainly in general pediatric populations, suggest that replacing sugar-containing foods and beverages with NNS-containing alternatives may modestly reduce short-term energy intake and attenuate weight gain, whereas long-term effects remain inconsistent. Observational studies frequently report positive associations between NNS consumption and higher BMI or adiposity, although these findings are likely explained by reverse causality, residual confounding, exposure misclassification, and methodological differences. Evidence in children with diabetes is scarce, heterogeneous, and rarely distinguishes between T1D and T2D. Proposed mechanisms include alterations in appetite regulation, reward processing, energy compensation, gut microbiota, and incretin and insulin responses, but human evidence remains inconsistent and does not demonstrate clinically meaningful long-term effects on body weight.

**Conclusion:**

Current evidence does not support a consistent or clinically meaningful long-term effect of NNS consumption on body weight in children with T1D or T2D. Although NNSs may reduce sugar intake and improve short-term glycaemic management, they should be viewed as adjunctive dietary tools rather than primary weight-management interventions. Future well-powered, long-term studies should evaluate individual sweeteners separately and distinguish between diabetes types to inform diabetes-specific dietary recommendations.

## 1 Introduction

Over the past several decades, the global prevalence of childhood obesity has increased dramatically, representing one of the most pressing public health challenges worldwide. According to the NCD Risk Factor Collaboration, the global prevalence of obesity among children and adolescents aged 5–19 years increased more than 10-fold between 1975 and 2016, while recent estimates indicate that over 390 million children and adolescents are currently overweight or obese worldwide (1, 2). This trend reflects complex interactions among dietary patterns, physical inactivity, urbanization, and profound changes in the food environment characterized by increased availability of foods and beverages that are energy-dense, highly palatable, rich in added sugars and saturated fats, and often low in dietary fiber, many of which are classified as ultra-processed foods (3, 4). Childhood obesity is of particular concern because it is associated not only with immediate metabolic complications but also with strong tracking into adulthood, increasing the risk of cardiovascular disease, type 2 diabetes, and premature mortality (5, 6).

Parallel to the increasing prevalence of childhood obesity, the incidence of both type 1 diabetes (T1D) and type 2 diabetes (T2D) has also risen worldwide, although these temporal trends reflect fundamentally different epidemiological drivers and disease mechanisms. While T1D remains the most common form of diabetes in pediatric populations, its incidence continues to increase globally at approximately 2–4% per year (7). Although the reasons for this increase are not fully understood, current evidence suggests that complex interactions between genetic susceptibility and environmental factors, rather than increasing adiposity, contribute to disease development. In contrast, the incidence of T2D—previously considered almost exclusively an adult-onset condition—has increased substantially in youth, particularly in association with rising obesity rates and socioeconomic disparities (8, 9). This shift concerning the incidence of T2D is largely driven by increasing adiposity, reduced physical activity, and early-life exposure to obesogenic environments (10). Importantly, the coexistence of obesity and T2D in childhood introduces additional metabolic complexity, as both conditions interact to worsening insulin resistance, glycemic instability, and overall cardiometabolic risk profiles (11, 12).

Consequently, body weight management has become an important component of pediatric diabetes care; however, its clinical objectives and underlying physiological determinants differ considerably between T1D and T2D. In T1D, intensive insulin therapy, although essential for optimal glycemic control and reduction of microvascular complications, is frequently associated with body weight gain (13, 14). This is mediated through multiple mechanisms, including insulin's anabolic effects on adipose and muscle tissue, suppression of lipolysis, reduced glycosuria, and improved metabolic efficiency leading to greater caloric retention (15). Furthermore, in children with T1D, several treatment-related factors such as defensive snacking to prevent hypoglycemia and overtreatment of hypoglycemic episodes can contribute to chronic positive energy balance (16, 17).

In contrast to T1D, where body weight gain is largely influenced by insulin therapy and its behavioral consequences, excess adiposity is a central pathogenic driver of T2D. More to the point, in children with T2D, excess adiposity is not only a key etiological factor but also a major driver of disease progression. Visceral fat accumulation contributes to systemic insulin resistance, chronic low-grade inflammation, and beta-cell dysfunction, creating a self-perpetuating cycle of worsening hyperglycemia and further weight gain (18, 19). Consequently, dietary strategies that simultaneously support glycemic control and promote healthy weight trajectories are of paramount importance in pediatric T2D management. However, implementing such strategies is challenging due to the need to balance energy restriction with adequate nutritional intake required for normal growth and pubertal development (16, 20).

Against this clinical background, non-nutritive sweeteners (NNSs), also referred to as artificial or low-calorie sweeteners, have emerged as widely used dietary substitutes for added sugars. These compounds provide a sweet taste with minimal or no caloric content and generally produce little or no direct increase in postprandial blood glucose concentrations when consumed as substitutes for added sugars. Howevr, physiological responses may vary according to the specific sweetener, dietary context, and individual metabolic status (21, 22). Accordingly, current diabetes guidelines indicate that NNSs may be used as substitutes for added sugars under appropriate dietary circumstances, including in children and adolescents with diabetes (23). Commonly used NNSs include aspartame, sucralose, saccharin, acesulfame potassium, and steviol glycosides, which are present in a broad range of food products such as diet beverages, sugar-free snacks, chewing gums, dairy products, and pharmaceutical preparations (24, 25). Their widespread incorporation into the modern food supply has led to near-ubiquitous exposure in some pediatric populations (26, 27).

The rationale for incorporating NNSs into dietary management is not that they exert intrinsic therapeutic effects but that replacing added sugars may reduce dietary sugar intake, lower glycemic load, and, under appropriate dietary circumstances, decrease total energy intake while maintaining palatability of foods and beverages (28, 29). This substitution strategy is theoretically attractive because it reduces intake of foods and beverages containing added sugars that contribute energy with limited nutritional value, without requiring complete restriction of sweet-tasting foods, which are highly preferred by children. Additionally, NNSs are expected to improve short-term glycemic control by reducing postprandial glucose excursions associated with sugar consumption (30). However, despite their widespread use, the impact of NNSs on body weight regulation remains controversial and biologically complex. Furthermore, interpretation of the available evidence is complicated by substantial methodological heterogeneity, including differences in study design, exposure assessment, follow-up duration, the specific sweeteners evaluated, and the frequent treatment of chemically distinct NNSs as a homogeneous class.

Randomized controlled trials (RCTs) generally suggest that replacing sugar-containing products with NNS-containing alternatives may modestly reduce energy intake and attenuate short-term body weight gain, particularly when substitution is complete and sustained (28, 31). In contrast, observational studies frequently report positive associations between NNS consumption and higher body mass index (BMI) or adiposity (32, 33). These apparently conflicting findings may partly reflect fundamental methodological differences between study designs, including reverse causality, residual confounding, exposure assessment, and the different causal questions addressed by observational studies and randomized controlled trials.

Interpretation of the available literature is further complicated by the fact that chemically distinct NNSs are frequently evaluated as a single exposure despite important differences in their physicochemical characteristics, metabolism, and potentially their biological effects. Moreover, many commercially available products contain combinations of multiple sweeteners, further increasing methodological heterogeneity and complicating comparisons across studies.

In addition to these methodological considerations, several biological and behavioral mechanisms have been proposed to explain how NNSs may influence body weight regulation, although direct evidence in humans remains limited. These mechanisms may contribute to variability in physiological responses, whereas reverse causality, residual confounding, exposure misclassification, and differences between observational studies and RCTs largely explain the inconsistent findings reported across the literature. These include disruption of learned associations between sweet taste and caloric content, altered reward processing, and changes in appetite regulation and satiety signaling (34, 35). Additional mechanisms include incomplete energy compensation and potential alterations in gut microbiota composition that may influence metabolic regulation (36, 37). Importantly, reverse causality is also a major concern in observational research, as children with overweight or obesity may be more likely to consume NNS-containing products, thereby confounding observed associations (38).

Children with diabetes represent a unique and understudied population in whom body weight regulation is influenced not only by growth and pubertal development but also by disease-specific metabolic and therapeutic factors. In T1D, exogenous insulin therapy and hypoglycaemia prevention strategies may influence energy balance, whereas in T2D, obesity, insulin resistance, and lifestyle modification remain central determinants of disease progression (16, 39). These factors may significantly modify both energy balance regulation and behavioral responses to sweet taste exposure.

Despite the rapidly expanding literature evaluating NNSs in general pediatric and adult populations, relatively few studies have specifically examined children with T1D or T2D, and even fewer have analyzed these conditions separately. Consequently, much of the current clinical guidance relies on indirect evidence extrapolated from populations with different metabolic characteristics, limiting the ability to draw definitive conclusions (16, 39). This lack of population-specific research hampers the development of precise, evidence-based dietary recommendations tailored to pediatric T1D and T2D care.

**Figure 1:**
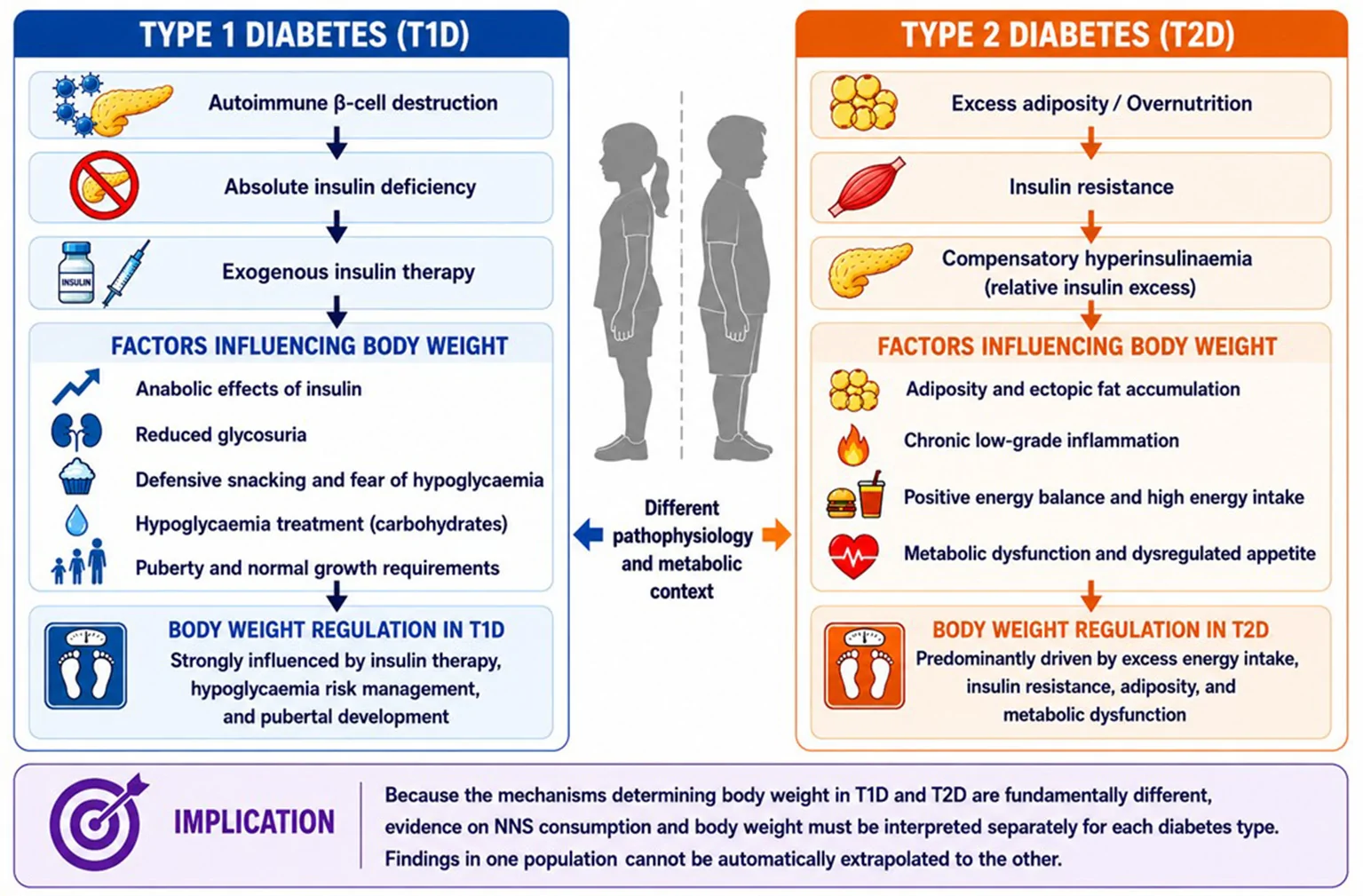
Body weight regulation in pediatric diabetes: Why T1D and T2D must be interpreted separately.

Although both T1D and T2D are characterized by chronic hyperglycemia, they differ markedly in disease pathophysiology, insulin physiology, body weight trajectories, and nutritional priorities. Consequently, the available evidence regarding NNSs and body weight should not be interpreted uniformly across diabetes types. Throughout this review, findings are presented separately for T1D and T2D whenever evidence allows, while also highlighting areas in which common dietary principles apply to both conditions. Figure 1 summarizes the distinct pathophysiological, metabolic, and therapeutic determinants of body weight regulation in children with T1D and T2D, illustrating why the potential effects of NNSs should be interpreted separately for each diabetes type throughout this review.

Therefore, the aim of this narrative review is to critically appraise and synthesize the current evidence regarding the relationship between NNS consumption and body weight in children with T1D and T2D, examine the proposed biological and behavioral mechanisms underlying these associations, discuss the dietary and clinical implications, and identify key methodological limitations and research priorities. Throughout the review, evidence relating to T1D and T2D is considered separately whenever possible to facilitate disease-specific interpretation of the available literature.

## 2 Methods

### 2.1 Literature search strategy

A comprehensive narrative literature search was conducted to identify relevant studies examining the relationship between NNSs and body weight in pediatric populations, with particular attention to children with diabetes. Electronic databases including PubMed, Scopus, Web of Science, and Google Scholar were systematically searched for articles published in English up to April 2026.

The literature search combined controlled vocabulary (where applicable) and free-text keywords related to NNSs, pediatric diabetes, and body weight. Search terms for NNS included both general terminology (e.g., non-nutritive sweeteners, artificial sweeteners, low-calorie sweeteners, non-caloric sweeteners, high-intensity sweeteners) and the names of individual sweeteners, including aspartame, sucralose, saccharin, acesulfame potassium (acesulfame-K), cyclamate, neotame, advantame, stevia, steviol glycosides, monk fruit (luo han guo), and mogrosides. These terms were combined with keywords related to type 1 diabetes, type 2 diabetes, pediatric diabetes, children, adolescents, body weight, obesity, overweight, adiposity, body mass index (BMI), body weight gain, and weight management using Boolean operators (AND/OR). The complete search strategies used for each database are provided in Supplementary Table S1.

Although this review is narrative rather than systematic, the search strategy was informed by established reporting frameworks such as PRISMA to enhance transparency and reproducibility (40). Reference lists of relevant articles and prior systematic reviews were manually screened to identify additional studies not captured in the initial search.

### 2.2 Inclusion criteria

Studies were included if they met the following criteria:

- Included pediatric populations (≤18 years), consistent with definitions used in pediatric clinical and epidemiological research
- Assessed intake or exposure to NNSs
- Reported outcomes related to body weight, including BMI, weight change, or adiposity measures
- Included study designs such as RCTs, cohort studies, case-control studies, cross-sectional studies, and relevant systematic reviews
- Included children with diabetes or populations with metabolic characteristics relevant to diabetes.

### 2.3 Exclusion criteria

Studies were excluded if they:

- Included only adult populations, unless providing mechanistic insights applicable to pediatric populations
- Focused exclusively on animal or *in vitro* models, except when used to support biological mechanisms
- Did not assess body weight or related outcomes
- Focused exclusively on nutritive sweeteners without comparison to NNS
- Were editorials, conference abstracts, or non-peer-reviewed reports

### 2.4 Study selection and synthesis approach

Given the narrative nature of this review, a formal systematic selection process was not applied. Instead, studies were selected based on relevance, methodological rigor, and contribution to the topic, consistent with recommended approaches for narrative reviews (41, 42).

Priority was given to:

- RCTs examining NNS substitution
- Prospective cohort studies evaluating long-term associations
- Systematic reviews and meta-analyses

Findings were synthesized qualitatively and organized into thematic sections, including evidence from general pediatric populations, studies involving children with diabetes, and mechanistic pathways. Where direct evidence in children with diabetes was lacking, findings from related populations were interpreted cautiously, in line with prior reviews in this field (43, 44).

### 2.5 Limitations of the Methodological Approach

As a narrative review, this study is subject to inherent limitations, including potential selection bias and lack of quantitative synthesis (41, 42). The heterogeneity of study designs, populations, and types of NNSs further limits comparability across studies (33). Additionally, the scarcity of studies specifically targeting children with diabetes represents a major limitation in the available evidence base, as highlighted in recent consensus statements and systematic reviews (16, 39).

## 3 Overview of non-nutritive sweeteners (NNSs)

### 3.1 Definition and classification

NNSs are sweetening agents that provide little or no metabolizable energy, while imparting an intensely sweet taste. Throughout this review, the term NNSs is used consistently to refer to these compounds. Although related terms such as high-intensity sweeteners, low-calorie sweeteners, and non-sugar sweeteners are used in the literature and in some clinical guidelines, these terms are not entirely synonymous and may reflect differences in regulatory definitions or emphasis (21, 45). These substances are characterized by a sweetness potency ranging from several hundred to several thousand times greater than that of sucrose, allowing their use in very small quantities while achieving the desired sensory profile (24). Owing to this high sweetness intensity, NNSs are incorporated into foods either individually or, more commonly, in blends to optimize sweetness perception, mask undesirable aftertastes, improve stability, and enhance sensory characteristics across different food matrices. Recent analyses of the food environment indicate that NNSs are increasingly incorporated into ultra-processed foods marketed to children, particularly beverages and “reduced-sugar” products, reflecting their expanding role in the global food supply (24, 27, 46).

NNSs can be broadly classified into two principal categories according to their origin: synthetic (artificial) and naturally derived sweeteners. Artificial sweeteners include compounds such as aspartame, sucralose, saccharin, and acesulfame potassium. These compounds have undergone extensive toxicological evaluation by regulatory authorities, including the European Food Safety Authority (47) and the U.S. Food and Drug Administration (48), both of which have established acceptable daily intake (ADI) values based on available safety data. Large-scale dietary surveillance studies in Europe and North America indicate that population-level intakes of approved NNSs generally remain below established ADIs, although exposure continues to increase, particularly among children and adolescents (45, 49).

Naturally derived NNSs include steviol glycosides, extracted from Stevia rebaudiana, and monk fruit extract (mogrosides), although the latter remains less commonly used in pediatric populations (18, 50). Importantly, individual NNSs differ considerably in their chemical structure, metabolic fate, sweetness potency, receptor interactions, and sensory profile. For example, aspartame provides approximately 200-fold greater sweetness than sucrose and is metabolized into amino acids and methanol, whereas sucralose is approximately 600-fold sweeter and is largely excreted unchanged (50). Saccharin is approximately 300–400 times sweeter than sucrose and is not metabolized in the human body (51). These physicochemical and metabolic differences may influence not only taste perception but also physiological responses. Indeed, emerging human studies suggest that individual sweeteners may differentially influence hormonal responses, glucose metabolism, and neural reward pathways, although current findings remain inconsistent (52, 53).

In addition to their use as individual sweeteners, NNSs are frequently combined within the same food product to enhance sweetness synergy, improve taste quality, and optimize product stability. Consequently, commercially available foods and beverages often contain multiple NNSs simultaneously rather than a single compound (54). Reformulation of sugar-sweetened beverages with NNSs has contributed to reductions in added sugar intake in some populations, particularly among children and adolescents (55, 56). However, the widespread incorporation of NNSs into commercially available foods has resulted not only in near-ubiquitous exposure among pediatric populations but also in increasing exposure to complex mixtures of different sweeteners.

From a research perspective, this heterogeneity has important methodological implications. Despite their substantial differences in chemical structure, metabolism, receptor interactions, sweetness potency, and potential biological effects, individual NNSs are frequently grouped together as a single exposure category in observational studies, RCTs, systematic reviews, and meta-analyses. Such aggregation may obscure compound-specific physiological effects, mask potential synergistic or interactive effects between sweeteners that are commonly consumed in combination, and contribute to the considerable clinical and statistical heterogeneity observed across studies.

Subsequently, conclusions regarding the health effects of “NNSs” should be interpreted cautiously, as they may not accurately reflect the biological properties of individual sweeteners. This represents one of the principal methodological limitations of the current evidence base and highlights the need for future investigations and evidence syntheses to evaluate individual sweeteners—or clearly defined sweetener combinations—separately whenever feasible.

### 3.2 Metabolic characteristics

A defining characteristic of NNSs is their minimal contribution to energy intake and limited direct effect on postprandial glycaemia. Unlike nutritive sweeteners such as sucrose or fructose, NNSs do not significantly increase blood glucose concentrations when consumed in isolation (30, 45). This glycemic neutrality has supported their widespread use as sugar substitutes in dietary strategies for diabetes management. Recent systematic reviews conducted in individuals with diabetes indicate that replacing sugars with NNSs can reduce acute glycemic excursions without adversely affecting short-term insulin responses (30, 57, 58).

The metabolic disposition of NNSs varies considerably according to the individual compound. Sucralose and saccharin are poorly absorbed and largely excreted unchanged, whereas aspartame is completely hydrolyzed into amino acids and methanol before entering normal metabolic pathways (59). Despite these metabolic differences, the quantities typically consumed are too small to contribute meaningfully to total energy intake.

NNSs are generally considered to exert minimal direct effects on insulin secretion when consumed alone. Nevertheless, increasing evidence suggests that their physiological actions may not be entirely metabolically inert. Controlled intervention studies and meta-analyses have reported inconsistent effects on incretin secretion, glycemic responses, and glucose metabolism, with findings varying according to the specific sweetener investigated, administered dose, co-ingestion with other nutrients, and underlying metabolic status of the study population (28, 60, 61). Similarly, experimental and clinical studies suggest that certain NNSs may influence gut microbial composition and glucose tolerance, although evidence in humans—and particularly in children—remains limited and inconsistent (36, 62, 63).

The concept of glycemic neutrality has therefore supported the inclusion of NNSs in dietary recommendations for diabetes. However, physiological responses appear to be influenced by multiple factors, including the timing of consumption, co-ingestion with carbohydrates, habitual dietary patterns, insulin sensitivity, gut microbiota composition, and individual metabolic phenotype (26, 28, 45). Importantly, these responses should not be assumed to apply uniformly across all NNSs. Individual sweeteners differ substantially in their absorption, metabolism, interaction with sweet taste receptors, gastrointestinal handling, and potential effects on incretin secretion, gut microbiota composition, neural reward pathways, and glucose homeostasis. Consequently, treating chemically distinct sweeteners as a single exposure category may obscure compound-specific physiological effects and represents an important source of heterogeneity within the current evidence base.

The above issue has important implications for the interpretation of clinical studies and evidence syntheses. Observational studies, RCTs, and meta-analyses that aggregate different NNSs into a single analytical category may inadvertently mask true sweetener-specific effects, increase clinical and statistical heterogeneity, and contribute to the inconsistent or inconclusive findings frequently reported in the literature. Furthermore, because commercially available products often contain combinations of multiple sweeteners, potential synergistic or interactive effects remain largely unexplored. Future mechanistic studies, clinical trials, and evidence syntheses should therefore evaluate individual sweeteners—or clearly defined sweetener combinations—separately whenever feasible to better characterize their distinct metabolic effects and provide more robust evidence for clinical and public health recommendations.

### 3.3 Consumption patterns in children with diabetes

Children with diabetes are routinely advised to limit added sugars as part of dietary management strategies aimed at optimizing glycemic control and reducing cardiometabolic risk (23, 64). Consequently, many children with diabetes and their families may choose NNS-containing products as substitutes for sugar-sweetened foods and beverages as part of dietary management. Although comprehensive dietary intake data are scarce in children with diabetes, national dietary surveys conducted in general pediatric and adolescent populations indicate that NNS consumption is particularly common through diet beverages and “sugar-free” packaged foods, with exposure increasing in recent years (30, 57, 65). Importantly, these survey findings should be regarded as indirect evidence because they describe NNS consumption patterns in general pediatric and adolescent populations rather than specifically in children with T1D or T2D. Consequently, extrapolation of these findings to pediatric diabetes should be undertaken cautiously until diabetes-specific dietary surveillance data become available (30, 57, 65).

Common sources of NNSs in the general pediatric population include diet soft drinks, flavored yogurts, desserts, chewing gums, and “no added sugar” processed foods. Within this population, replacing sugar-sweetened beverages with NNS-containing alternatives has been evaluated as a potential strategy to reduce energy intake (30, 31). RCTs conducted predominantly in general pediatric populations suggest that replacing sugar-sweetened beverages with NNS-containing alternatives may modestly reduce energy intake and attenuate weight gain over time (66, 67). However, interpretation of the overall evidence remains challenging because studies differ considerably with respect to intervention duration, comparator beverages, adherence, and the specific NNSs evaluated. Furthermore, findings from observational studies should be interpreted separately, as they are additionally influenced by reverse causality, residual confounding, and exposure misclassification. Whether these intervention findings can be generalized to children with T1D or T2D remains uncertain because comparable diabetes-specific randomized controlled trials evaluating NNS consumption and body weight are scarce.

Consumption patterns among children with diabetes are shaped not only by general dietary preferences but also by disease-specific nutritional management. In children with T1D, NNS-containing foods and beverages are commonly selected to reduce the glycemic impact of dietary sugars, while maintaining dietary flexibility and facilitating carbohydrate counting within intensive insulin therapy (16, 64). Sugar-free beverages, chewing gums, desserts, and confectionery products are frequently incorporated into everyday eating patterns as alternatives to sugar-containing products, although they should not be regarded as substitutes for the rapid-acting carbohydrates required for the treatment of hypoglycaemia (23, 64). In contrast, among children and adolescents with T2D, the use of NNS-containing products is generally encouraged within broader lifestyle interventions aimed at reducing added sugar intake, lowering dietary energy density, and supporting body weight management (23, 39). Nevertheless, despite these differences in clinical rationale, robust data describing the frequency, quantity, and long-term patterns of NNS consumption in children with either T1D or T2D remain remarkably limited. Consequently, current understanding of diabetes-specific NNS exposure continues to rely largely on extrapolation from studies conducted in general pediatric populations.

In both general pediatric populations and children with diabetes, consumption patterns are influenced by multiple factors, including age, parental feeding practices, socioeconomic status, and food marketing exposure (32, 68, 69). Adolescents tend to have higher consumption due to increased autonomy and exposure to marketing, whereas younger children's intake is more influenced by parental choices (68). Notably, among younger children, parental purchasing decisions and household dietary practices largely determine exposure to NNS-containing products, whereas adolescents progressively make independent food choices and therefore experience greater exposure through school environments, restaurants, convenience foods, and marketing (32, 68, 69).

Evidence from general pediatric populations suggests that children with overweight or obesity may be more likely to consume NNS-containing products as part of body weight-management strategies, raising concerns about reverse causality in observational studies (28, 38). Similar patterns may also occur among children with T2D, although diabetes-specific evidence remains limited.

Accurate quantification of NNSs intake remains challenging. Dietary assessment tools often lack specificity regarding type and quantity of sweeteners, and labeling practices may not fully disclose NNSs content (26, 70). Validation studies indicate that traditional dietary recall methods may underestimate exposure compared with biomarker-based approaches (70, 71).

**Figure 2:**
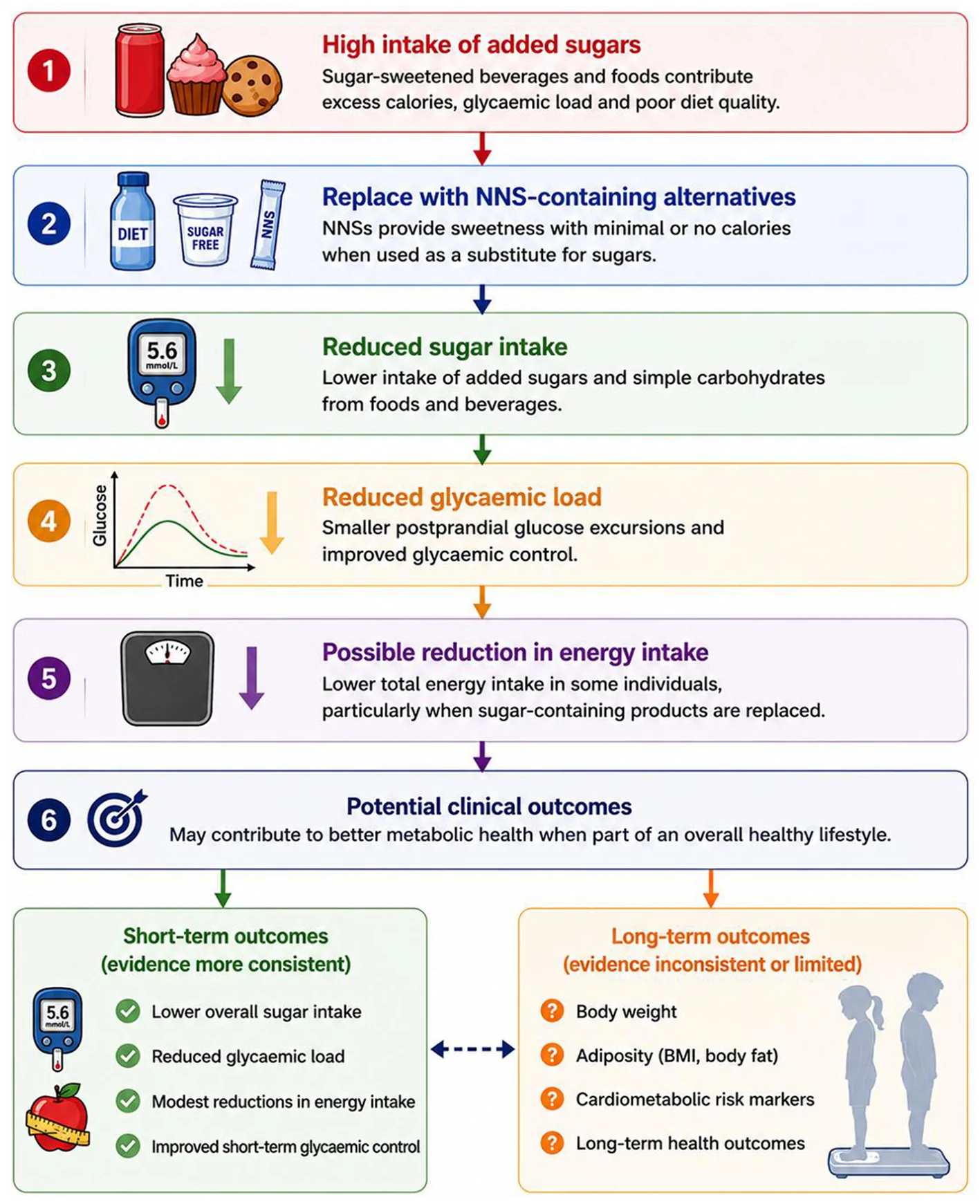
Clinical pathway of NNS use in children with diabetes.

Overall, while NNSs are widely integrated into pediatric diabetes management, their consumption patterns are highly heterogeneous and influenced by complex behavioral, environmental, clinical, and commercial factors. Moreover, because children are frequently exposed to multiple sweeteners simultaneously through processed foods and beverages, accurately characterizing exposure to individual NNSs remains challenging. This limitation should be carefully considered when interpreting epidemiological studies and developing future dietary recommendations. Furthermore, much of the current understanding of NNS consumption patterns in pediatric diabetes continues to rely on evidence extrapolated from general pediatric populations, highlighting the need for diabetes-specific dietary surveillance studies and prospective observational research. The proposed role of NNSs within pediatric diabetes dietary management is summarized in Figure 2, illustrating the theoretical clinical pathway through which replacing added sugars with NNS-containing foods and beverages may reduce glycemic load and, under appropriate dietary circumstances, decrease energy intake, while highlighting the uncertainty surrounding long-term body weight outcomes.

## 4 Body weight regulation in children with diabetes

### 4.1 Unique considerations

**Figure 3:**
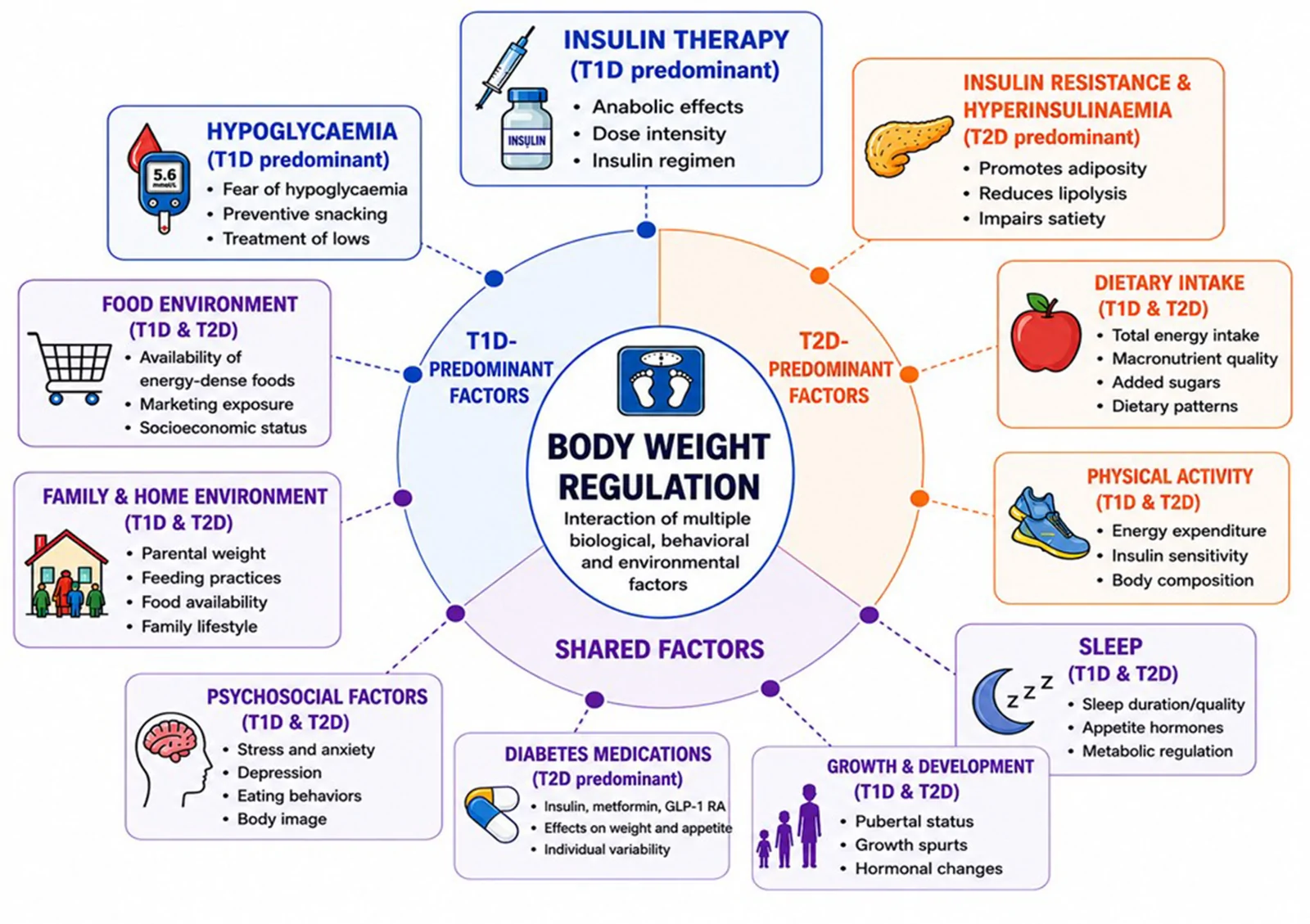
Determinants of body weight regulation in children with diabetes.

Body weight regulation in children with diabetes is influenced by a complex interplay of disease-specific, hormonal, metabolic, behavioral, and treatment-related factors that distinguish this population from healthy peers (10, 72, 73). In addition to energy intake and physical activity, glycemic variability, insulin dynamics, and developmental stage all contribute to body composition changes over time (64, 74). The principal biological, treatment-related, behavioral, developmental, and environmental determinants influencing body weight regulation in children with diabetes are summarized in Figure 3. These factors interact dynamically and differ in their relative importance between T1D and T2D because of fundamental differences in disease pathophysiology, insulin physiology, and therapeutic management.

In T1D, the initiation and intensification of insulin therapy are strongly associated with body weight gain (13, 23). This effect is primarily driven by the anabolic properties of insulin, which promotes glucose uptake in skeletal muscle and adipose tissue, enhances lipogenesis, stimulates protein synthesis, and suppresses lipolysis (74, 75). Prior to diagnosis, children with T1D often experience body weight loss due to absolute insulin deficiency, leading to unopposed catabolic processes including increased lipolysis, proteolysis, and glycosuria (64, 76). Following insulin initiation, restoration of metabolic control reverses these catabolic pathways, resulting in rapid body weight regain and, in some cases, overshoot body weight gain (64, 77).

Reduced glycosuria following insulin therapy also leads to improved caloric efficiency, increasing net energy retention even without dietary changes (20, 64, 74). This “metabolic recovery effect” is increasingly recognized as an important contributor to early BMI rebound after diagnosis.

Intensive insulin regimens, including multiple daily injections and continuous subcutaneous insulin infusion, are associated with improved glycemic control but may also increase body weight gain risk due to tighter correction of hyperglycemia and more flexible eating patterns (23, 74, 78). Episodes of hypoglycemia are a key behavioral driver of increased energy intake, as children are frequently advised to consume rapid-acting carbohydrates to correct low blood glucose (64, 79). This can lead to repeated “defensive eating,” particularly when hypoglycemia is frequent or overtreated (80, 81).

Fear of hypoglycemia may further contribute to intentional maintenance of higher glucose levels or pre-emptive snacking, both of which promote chronic positive energy balance (82). Recent pediatric studies using continuous glucose monitoring suggest that improvements in time-in-range may be accompanied by compensatory increases in caloric intake in some individuals (74, 82, 83).

In T2D, excess body weight is typically a central etiological factor rather than a consequence of treatment (23). Pediatric T2D is strongly associated with obesity, visceral adiposity, and insulin resistance, often coexisting with dyslipidemia, hypertension, and non-alcoholic fatty liver disease (84, 85). Insulin resistance leads to compensatory hyperinsulinemia, which promotes adipocyte lipid storage and further weight gain, reinforcing a self-perpetuating metabolic cycle (86, 87).

The coexistence of obesity and diabetes influences body weight regulation differently according to diabetes type. In children with T2D, excess adiposity, insulin resistance, and metabolic dysregulation reinforce one another in a self-perpetuating cycle. In contrast, in children with T1D—and in youth with T2D receiving insulin therapy—body weight gain is more strongly influenced by the anabolic effects of exogenous insulin than by obesity-driven insulin resistance (85, 88). Pharmacological treatments may also influence body weight trajectories. Insulin therapy is associated with body weight gain, whereas glucagon-like peptide-1 receptor agonists (GLP-1 RAs), including liraglutide and semaglutide, have demonstrated clinically meaningful body weight reduction in adolescents with obesity and improvements in both body weight and glycemic control in adolescents with T2D (89, 90). However, long-term pediatric safety and real-world effectiveness data remain limited (89, 91).

Both T1D and T2D in children are further influenced by pubertal hormonal changes, including increases in growth hormone and sex steroids, which transiently reduce insulin sensitivity and alter body composition (92). This pubertal insulin resistance contributes to increased fat mass deposition and makes weight management particularly challenging during adolescence (64, 93).

### 4.2 Dietary management challenges

Managing body weight in children with diabetes requires balancing optimal glycemic control with adequate energy and nutrient intake to support normal growth, neurodevelopment, and pubertal maturation (93, 94). Unlike adults, children require dynamic adjustment of energy and nutrient intake according to age, growth velocity, physical activity, and pubertal stage, making overly restrictive dietary approaches potentially harmful if not carefully supervised (93, 94).

A cornerstone of dietary management in pediatric diabetes is individualized carbohydrate management, which encompasses not only limiting free sugars and high-glycemic index foods, but also consideration of total carbohydrate amount, carbohydrate quality, dietary fiber intake, meal timing and composition, and appropriate matching of carbohydrate intake with insulin therapy, particularly in children with T1D (16, 95). Within this framework, many children and adolescents use commercially available “reduced-sugar” or “sugar-free” products containing non-nutritive sweeteners (NNSs) as substitutes for sugar-containing foods and beverages (96, 97). Importantly, the replacement of added sugars with NNS-containing alternatives is not inherently problematic and may reduce acute glycemic load and, under appropriate dietary circumstances, decrease energy intake when these products replace, rather than are consumed in addition to, sugar-containing foods or beverages (28, 30, 31). This conceptual pathway is illustrated in Figure 2 and provides the clinical rationale underlying current dietary recommendations for the use of non-nutritive sweeteners in pediatric diabetes. Rather, concerns relate to the possibility that frequent reliance on commercially available ultra-processed “reduced-sugar” products may be associated with displacement of nutrient-dense foods, reinforcement of preferences for highly sweet-tasting foods, “health-halo” perceptions that may encourage compensatory intake or larger portion sizes, and poorer overall dietary pattern quality, although current evidence supporting these mechanisms remains limited and causal relationships have not been established (3, 26, 98). Emerging evidence also suggests that repeated exposure to intense sweetness may influence appetite regulation, sweet taste conditioning, and reward pathways; however, human evidence remains inconclusive, particularly in children with diabetes (35).

Flexibility in dietary patterns is essential to enable appropriate matching of insulin dosing with food intake and physical activity. Carbohydrate counting remains a cornerstone of nutritional management, particularly in T1D, but requires advanced numeracy skills, sustained family engagement, and continuous education (16, 86). Even minor inaccuracies in carbohydrate estimation or insulin dosing can lead to glycemic variability, which in turn may influence subsequent eating behavior and compensatory energy intake (99).

Behavioral factors play a central role in determining adherence to dietary recommendations and long-term body weight regulation in children with diabetes. Sustaining dietary changes over time can be challenging because food choices are influenced not only by nutritional knowledge but also by taste preferences, eating behaviors, psychosocial factors, and the surrounding food environment. Children and adolescents with diabetes may exhibit increased cognitive restraint and heightened preferences for sweet-tasting foods, particularly in the context of dietary restrictions and intensive dietary self-management, which may promote compensatory eating behaviors (100). Furthermore, the widespread availability and marketing of “sugar-free” or NNS-containing products may contribute to a “health-halo” effect, whereby these products are perceived as inherently healthier and therefore consumed in larger quantities or with reduced attention to portion size (98).

Psychosocial determinants—including family dynamics, parental support, peer influence, diabetes-related distress, and developmental stage—also have an important impact on adherence to dietary recommendations. Among children with diabetes, adolescents are particularly vulnerable to dietary non-adherence because of increasing autonomy, social influences, and psychosocial stressors (101). In children with T1D, disordered eating behaviors, including intentional insulin omission, represent an additional and clinically important challenge because they are associated with poor metabolic control and increased risk of diabetes-related complications (102).

Environmental and socioeconomic influences further shape dietary behaviors. Food insecurity, school food environments, cultural norms, and aggressive marketing of “diet” products all influence food choices and dietary quality (3, 68). Public health analyses further indicate that NNS-containing foods are increasingly incorporated into ultra-processed dietary patterns consumed by children and adolescents worldwide (3, 26).

Finally, long-term adherence to dietary recommendations remains one of the greatest challenges in pediatric diabetes management. Effective nutritional strategies should therefore be individualized, developmentally appropriate, culturally sensitive, and family-centered, emphasizing sustainable dietary patterns rather than short-term dietary restriction (103, 104). This complexity underscores the importance of evaluating NNS-containing products within the context of the overall dietary pattern, rather than considering them in isolation, while recognizing the distinct metabolic and therapeutic considerations that characterize T1D and T2D (103, 104).

Taken together, these considerations highlight that body weight regulation and dietary management differ substantially between children with T1D and those with T2D owing to fundamental differences in disease pathophysiology, insulin physiology, therapeutic goals, and the determinants of energy balance. Consequently, the potential effects of NNSs on body weight should be interpreted within the specific clinical context of each diabetes type rather than assuming a uniform effect across pediatric diabetes. These interacting determinants are summarized in Figure 3 and provide the conceptual framework for interpreting the evidence presented in the following sections. The following sections therefore critically examine the available evidence while distinguishing, whenever possible, between findings relevant to T1D and T2D.

## 5 Evidence on NNS and body weight in children

### 5.1 Differences between type 1 and type 2 diabetes: implications for interpreting the evidence

Although both T1D and T2D require careful dietary management and glycemic control, they differ substantially in their underlying pathophysiology, insulin physiology, body weight regulation, and therapeutic priorities. These disease-specific differences, summarized in Figure 1, provide the conceptual framework for interpreting the available evidence on NNSs and body weight throughout this section.

In T1D, body weight is strongly influenced by exogenous insulin therapy, which promotes anabolic metabolism and reduces glycosuria-related caloric loss. Hypoglycaemia treatment, insulin titration, and pubertal growth further dominate energy balance, meaning that dietary effects of NNS substitution are likely to be relatively modest compared with insulin-related determinants of body weight (17, 105).

In contrast, T2D is characterized by obesity and insulin resistance, making dietary composition considerably more relevant to energy balance regulation (106, 107). NNS substitution could theoretically reduce energy intake and support body weight management; however, compensatory eating, altered reward responses, and clustering with ultra-processed food consumption may attenuate these benefits (106, 107).

Current evidence remains insufficient to determine whether NNSs exert neutral, beneficial, or adverse effects on long-term weight outcomes in pediatric T2D (38, 106). Overall, heterogeneity in insulin physiology, disease mechanisms, and baseline adiposity suggests that NNS effects are context-specific, but robust comparative pediatric diabetes data remain lacking.

### 5.2 Evidence from general pediatric populations

Because RCTa evaluating the effects of NNSs on body weight in children with T1D or T2D are extremely limited, the available evidence is derived predominantly from general pediatric populations without diabetes. Consequently, the findings summarized in this section should be interpreted cautiously when considering pediatric diabetes, as body weight regulation, metabolic characteristics, and therapeutic goals differ substantially between children without diabetes and those with T1D or T2D. Studies in general pediatric populations provide inconsistent and context-dependent findings regarding the relationship between NNS consumption and body weight. The majority of evidence comes from RCTs, prospective cohort studies, and cross-sectional analyses, each yielding different interpretations (31, 33, 38, 97, 108). In addition to differences in study design, heterogeneity in NNS type, dose, exposure duration, and dietary background further contributes to variability in findings (38, 108).

RCTs conducted primarily in school-aged children from the general population, including children with overweight or obesity but not specifically children with T1D or T2D, generally suggest that replacing sugar-sweetened beverages with NNS-containing alternatives may lead to modest reductions in weight gain or BMI increase over time. Intervention studies demonstrate that substitution of caloric beverages with diet alternatives can reduce total energy intake and attenuate weight gain trajectories, particularly in overweight or high-risk children (66, 97, 109, 110). These trials primarily evaluated body weight, BMI trajectories, and energy intake rather than glycemic control or diabetes-specific metabolic outcomes. Collectively, these findings support the hypothesis that NNSs may be beneficial when used as a direct replacement strategy for added sugars rather than as an addition to the habitual diet (28, 31, 38, 66, 97, 108). Some trials also indicate greater effectiveness when substitution is combined with broader behavioral or family-based dietary interventions (97, 109, 110).

However, the magnitude of these effects is generally small, and not all trials demonstrate statistically significant differences in long-term adiposity outcomes (38, 97). Many interventions are limited by relatively short follow-up durations, which may be insufficient to capture sustained effects on growth, puberty, and long-term energy balance (109, 111). Declining adherence and dietary compensation through other energy sources may further attenuate observed benefits (34, 38, 108). Because these intervention studies were conducted in children without diabetes, they provide little information regarding glycemic control, insulin requirements, or other diabetes-specific outcomes and therefore cannot be directly extrapolated to pediatric T1D or T2D populations (38, 97, 108).

Extrapolation of findings from general pediatric populations to children with diabetes should therefore be undertaken cautiously. In children with T1D, insulin therapy, insulin dose adjustment, and hypoglycaemia-related compensatory eating may substantially influence body weight regulation, whereas baseline adiposity, insulin resistance, and lifestyle-related factors are more relevant determinants of body weight in children with T2D. These diabetes-specific factors are generally not captured in studies conducted in children without diabetes.

In contrast, observational studies frequently report positive associations between NNSs intake and higher body weight, BMI, or adiposity markers in children (38, 97, 112). These associations should be interpreted cautiously due to methodological limitations. A key concern is reverse causality, whereby children who are already overweight may preferentially consume NNSs-containing products as part of weight-control behaviors (38, 108, 112).

Additional sources of bias include residual confounding from dietary quality, physical activity, sleep, parental adiposity, and socioeconomic status (33, 38, 108, 112). Furthermore, dietary assessment tools often lack precision in quantifying NNS exposure, particularly in ultra-processed foods, leading to exposure misclassification (3, 113, 114). Biomarker-based validation remains limited in pediatric populations (115, 116).

Overall, systematic reviews and meta-analyses conclude that current evidence from general pediatric populations remains inconsistent and insufficient to establish a clear causal relationship between NNSs intake and long-term body weight outcomes in children. Many conclude that observed associations are more consistent with residual confounding and reverse causality than with direct biological effects (33, 38, 97, 108). Importantly, these findings should not be assumed to apply directly to children with T1D or T2D, as diabetes-specific clinical trials evaluating the effects of NNSs on body weight remain extremely limited.

### 5.3 Evidence in children with diabetes

Evidence from clinical trials involving children with diabetes remains extremely limited, representing a significant research gap (117, 118). Existing studies are generally small, heterogeneous, and short-term and are often embedded within broader dietary or diabetes education interventions rather than specifically designed to evaluate the effects of NNSs on body weight (38, 117, 119).

Indirect evidence from short-term intervention studies suggests that replacing added sugars with NNS-containing foods or beverages may reduce short-term energy intake and, in some settings, attenuate postprandial glycemic excursions; however, these findings are derived largely from studies conducted outside pediatric diabetes populations (33, 38). However, these metabolic benefits have not consistently translated into clinically meaningful reductions in body weight or BMI over time (38, 108).

Interpretation of these findings should consider the important differences between T1D and T2D. In T1D, weight trajectories are strongly influenced by exogenous insulin therapy, hypoglycemia treatment, pubertal development, and insulin titration (17, 74). These factors may mask any modest contribution of NNSs to body weight regulation. Conversely, in T2D, obesity and insulin resistance are primary drivers of disease progression, suggesting that NNSs may have greater theoretical relevance for weight management. However, direct evidence in pediatric T2D remains extremely limited.

In children with T1D, the primary rationale for using NNSs is to reduce the glycemic impact of dietary sugars while preserving dietary flexibility and facilitating carbohydrate counting within intensive insulin therapy. Because body weight in T1D is largely determined by exogenous insulin administration and its associated metabolic and behavioral consequences, the independent contribution of NNSs substitution to long-term body weight regulation is likely to be relatively modest (17, 23, 64, 74, 105). Consequently, although replacing sugar-containing foods and beverages with NNSs-containing alternatives may modestly reduce acute glycemic excursions or short-term energy intake, current evidence does not support a clinically meaningful effect on long-term body weight. Future intervention studies in pediatric T1D should therefore evaluate body weight outcomes while carefully accounting for insulin regimen, total daily insulin dose, glycemic variability, frequency of hypoglycaemia, and pubertal status, all of which substantially influence energy balance and body composition (16, 39, 74).

In contrast, in children and adolescents with T2D, dietary interventions play a central role in disease management because obesity, excess adiposity, and insulin resistance are fundamental features of the disease (23, 85, 106, 107). Within this context, replacing added sugars with NNS-containing foods and beverages may reduce total energy intake and support body weight management when these products replace, rather than are consumed in addition to, sugar-containing foods or beverages and when the reduction in energy intake is not offset by compensatory increases in energy consumption from other dietary sources as part of comprehensive lifestyle interventions. Nevertheless, direct clinical evidence in pediatric T2D remains extremely limited, and much of the available knowledge is extrapolated from studies involving overweight or obese children without diabetes or mixed pediatric populations (38, 108). Consequently, adequately powered, long-term RCTs specifically enrolling children with T2D are required to determine whether replacing added sugars with NNSs-containing products provides clinically meaningful benefits for body weight regulation, glycaemic control, and long-term cardiometabolic health.

Observational studies in children with diabetes are likewise scarce and methodologically limited. Existing studies report heterogeneous or non-significant associations between NNSs intake and BMI or adiposity (97, 120). A major limitation is indication bias, whereby children with obesity or poor metabolic control are more likely to receive dietary advice encouraging NNSs consumption (121). Additional confounding arises from differences in insulin regimens, carbohydrate counting, dietary counseling, physical activity, disease severity, and psychosocial factors, including diabetes distress (17, 74). In addition, NNS-containing products are frequently consumed within broader dietary patterns that differ in overall dietary quality and degree of ultra-processed food consumption. Therefore, any observed associations between NNS intake and body weight may partly reflect these broader dietary pattern characteristics rather than effects attributable to NNSs themselves (3, 97, 108).

Due to the scarcity of direct evidence, studies involving overweight and metabolically at-risk pediatric populations are often used as indirect evidence. These investigations report associations between NNSs consumption and higher BMI, waist circumference, and markers of insulin resistance (38, 97, 108). Nevertheless, these populations differ substantially from children with diabetes, particularly regarding insulin physiology, treatment strategies, and metabolic regulation, and therefore extrapolation should be made cautiously.

Overall, current evidence remains insufficient to determine whether NNSs exert beneficial, neutral, or adverse effects on long-term body weight in children with either T1D or T2D. Furthermore, many pediatric studies either combine participants with different diabetes types or fail to specify the diabetes type studied, thereby limiting diabetes-specific conclusions. Future investigations should therefore stratify participants according to diabetes type to provide clinically meaningful dietary recommendations.

## 6 Proposed biological mechanisms potentially linking NNSs to intermediate metabolic outcomes and body weight regulation

**Figure 4:**
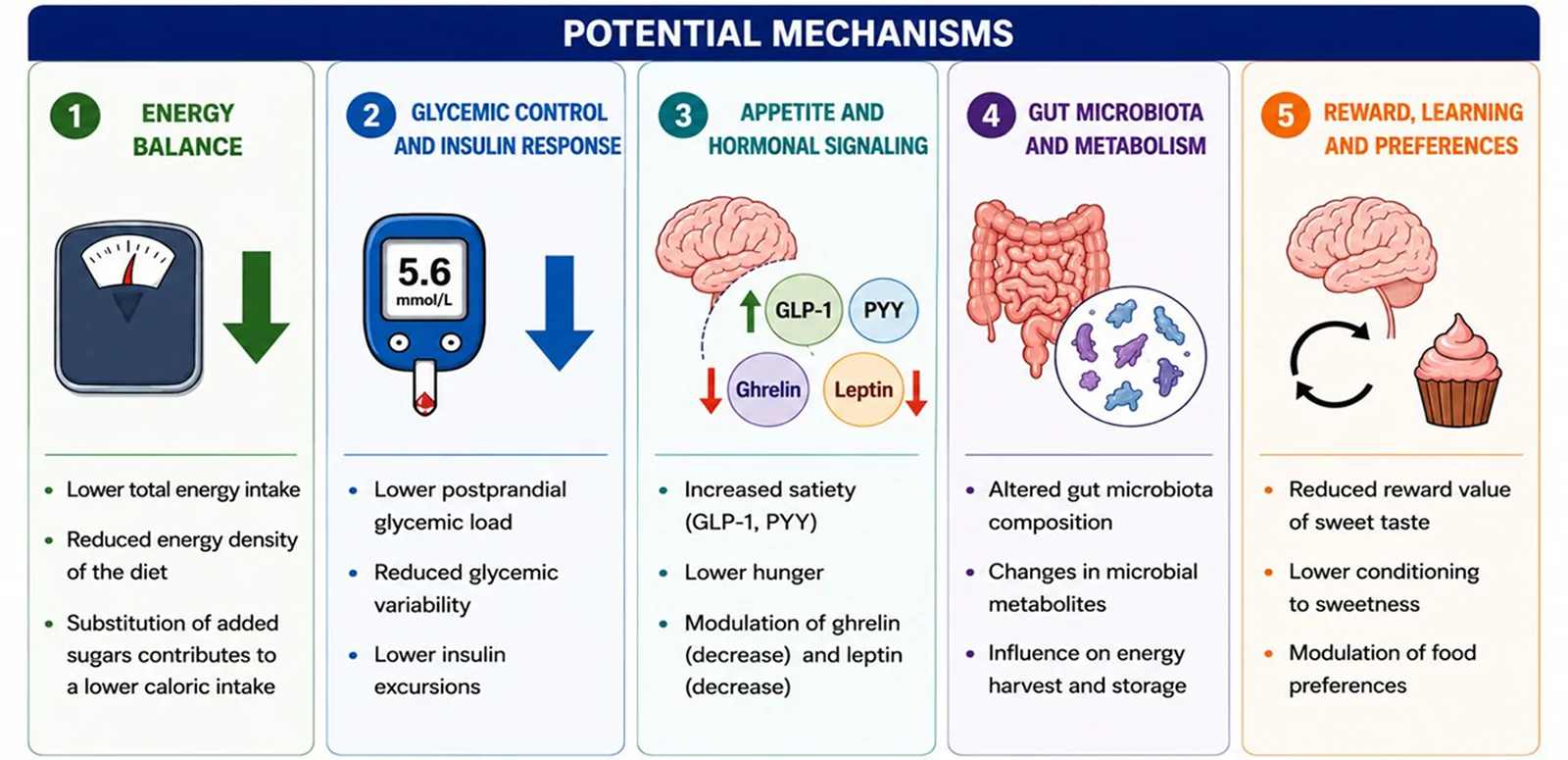
Proposed biological mechanisms linking NNSs to intermediate physiological outcomes.

Because current clinical evidence does not demonstrate a consistent long-term effect of NNS consumption on body weight in children with diabetes, the biological pathways discussed in this section should be regarded as proposed or hypothesized mechanisms rather than established explanations for changes in body weight. Most mechanistic evidence originates from studies in adults, general pediatric populations, animal models, or *in vitro* investigations, whereas direct mechanistic evidence in children with T1D or T2D remains extremely limited. Consequently, these pathways are presented as biologically plausible mechanisms that may influence intermediate physiological processes—including appetite regulation, energy intake, gut microbiota composition, and glycemic responses—which could potentially affect body weight under certain circumstances. Their relative contribution to long-term body weight regulation in children with diabetes remains uncertain and should be interpreted separately for T1D and T2D because of their distinct pathophysiology, insulin physiology, therapeutic approaches, and determinants of energy balance. The principal biological mechanisms through which non-nutritive sweeteners may influence body weight in children with diabetes are summarized in Figure 4. These interconnected pathways include effects on energy balance, glycemic and insulin responses, appetite and satiety signaling, gut microbiota composition and function, and reward-related eating behaviors.

### 6.1 Proposed effects on appetite regulation and satiety

Most evidence supporting this mechanism derives from adult human studies, experimental animal models, and in vitro investigations. Direct evidence in children with diabetes is extremely limited.

NNSs provide sweetness without caloric load, creating a potential dissociation between sweetness perception and post-ingestive energy delivery. This mismatch has been hypothesized to disrupt learned associations between sweet taste and energy intake established early in life (122, 123). Emerging longitudinal and developmental evidence suggests that early-life exposure to intense sweetness may shape long-term taste preferences and dietary patterns, potentially influencing obesity risk (123–125).

From a neurobiological perspective, sweet taste activates dopaminergic reward pathways involving regions such as the nucleus accumbens and orbitofrontal cortex. Experimental studies suggest that when sweetness is repeatedly experienced without the expected caloric reinforcement, reward prediction signaling may be altered, potentially reducing the expected post-ingestive reward from sweet taste and thereby modifying food reward, sweet taste preference, and food-seeking behavior. Consequently, some investigators have hypothesized that these neurobiological changes could either reduce or enhance subsequent food intake depending on the behavioral and dietary context; however, available human evidence remains inconsistent. Whether these changes increase or decrease subjective satiety or subsequent energy intake remains uncertain, as human studies have reported inconsistent findings (123, 126). Functional MRI studies in adults and adolescents similarly suggest that repeated exposure to non-caloric sweet taste may alter neural responses to sweet stimuli, although the direction and clinical significance of these changes remain inconsistent and context-dependent (127).

Sweet taste receptors (T1R2/T1R3), expressed in both oral and gastrointestinal tissues, have also been proposed to influence appetite regulation and gut hormone secretion (128). Experimental studies suggest that activation of these receptors by certain NNSs may influence the secretion of incretin hormones such as glucagon-like peptide-1 (GLP-1) and peptide YY (PYY), hormones that normally promote satiety and slow gastric emptying (128). Depending on the specific sweetener and experimental conditions, both increases and minimal or absent responses have been reported. However, the magnitude and even the direction of these hormonal responses vary according to the specific sweetener, dose, co-ingested nutrients, and metabolic status, with several controlled human studies reporting minimal or no clinically meaningful effects. Some studies also suggest differential responses among compounds such as sucralose, saccharin, and steviol glycosides (70).

Evidence in children remains limited. Appetite regulation is still developing and is highly sensitive to environmental influences and learned eating behaviors. Consequently, repeated exposure to intense sweetness has been hypothesized to reinforce preferences for sweet-tasting foods independent of caloric content (129, 130). However, whether these developmental mechanisms translate into clinically meaningful differences in long-term body weight remains uncertain.

From a diabetes-specific perspective, the potential relevance of these mechanisms may differ between T1D and T2D. In T1D, appetite and eating behavior are strongly influenced by insulin dosing, hypoglycaemia prevention and treatment, and carbohydrate replacement, factors that may overshadow any modest influence of NNSs on appetite regulation. Conversely, in T2D, where obesity, insulin resistance, and excess energy intake are central disease characteristics, alterations in appetite regulation, if present, could theoretically have greater relevance for energy intake and body weight regulation. However, direct evidence supporting this hypothesis in children with T2D is currently lacking. Despite the existence of several biologically plausible mechanisms (Figure 4), current human evidence has not consistently demonstrated clinically meaningful long-term effects of NNSs on body weight, highlighting the need to interpret mechanistic findings alongside data from intervention and observational studies.

### 6.2 Proposed effects on energy intake and energy compensation

Evidence is derived primarily from RCTs and controlled feeding studies in adults and general pediatric populations. Diabetes-specific pediatric evidence is scarce.

One of the principal hypotheses underlying NNS use is that replacing sugar-containing foods and beverages with NNS-containing alternatives may reduce total energy intake. Short-term randomized controlled trials conducted primarily in adults and, to a lesser extent, in children and adolescents suggest that replacing sugar-sweetened beverages with NNS-containing alternatives can acutely reduce caloric intake and modestly improve short-term weight-related outcomes (108).

However, long-term energy balance is regulated by multiple behavioral and physiological mechanisms. Experimental studies and controlled feeding trials conducted predominantly in adults indicate that reductions in energy intake following substitution of sugar with NNSs may be partially offset by compensatory increases in energy intake from other dietary sources (28). Importantly, incomplete energy compensation indicates that individuals do not fully replace the calories saved through sugar substitution and therefore still achieve a net reduction in energy intake. Nevertheless, over longer periods, partial compensatory eating, reduced adherence to dietary substitutions, and broader changes in dietary behavior may attenuate the initial energy deficit and thereby reduce the likelihood that short-term reductions in energy intake translate into sustained body weight loss (31, 131).

Additional behavioral mechanisms, identified primarily in adult and adolescent populations, have also been proposed. The “health halo” or licensing effect associated with low-calorie foods may encourage greater energy intake from other foods (132). Experimental studies conducted in adults and adolescents suggest that these cognitive biases may influence eating behavior in both adults and adolescents (132).

Some controlled feeding studies performed primarily in adults have suggested that satiety responses following NNS consumption may differ from those observed after caloric sweeteners, although findings remain heterogeneous and context-dependent (33).

The potential importance of these mechanisms may also differ according to diabetes type. In T1D, compensatory caloric intake frequently occurs as part of routine hypoglycaemia treatment or insulin dose adjustment. In contrast, in T2D, compensatory increases in energy intake could theoretically reduce the effectiveness of dietary strategies designed to lower excess adiposity and improve insulin sensitivity. Nevertheless, direct evidence demonstrating that these mechanisms produce clinically meaningful differences in body weight among children with diabetes is currently unavailable.

### 6.3 Proposed effects on gut microbiota

Most evidence derives from animal models and adult human studies. Pediatric evidence, particularly in children with diabetes, remains very limited.

The gut microbiota has been proposed as one of the potential biological mediators through which NNS consumption may influence metabolic regulation. Animal studies and translational research suggest that certain NNSs may alter microbial composition and function, potentially influencing energy harvest, short-chain fatty acid production, and host metabolism (36, 133).

Landmark experimental studies demonstrated that saccharin-induced alterations in the gut microbiome of mice impaired glucose tolerance, with these effects transferable through fecal transplantation (36). Subsequent investigations have explored similar mechanisms for other sweeteners, including sucralose and aspartame, although findings remain inconsistent and appear to depend on the specific sweetener, dose, and host characteristics (62, 134).

Evidence in humans remains heterogeneous. While some RCTs report minimal microbiome alterations following NNS consumption, others describe modest changes in microbial diversity without clear clinical significance (30, 62). A recent systematic review identified marked inter-individual variability as one of the principal limitations of the current evidence base (134, 135).

Evidence in children, and especially children with diabetes, remains scarce. Because the pediatric gut microbiome undergoes continuous maturation and is influenced by diet, medications, and developmental factors, isolating the specific contribution of NNSs is challenging (136). Consequently, microbiota-mediated effects on body weight remain biologically plausible but unproven in pediatric diabetes.

Moreover, the potential clinical significance of microbiome alterations may differ between T1D and T2D. In T1D, microbiota-related mechanisms have primarily been investigated in relation to immune regulation and autoimmunity, whereas in T2D they have been studied mainly in relation to obesity, insulin resistance, and metabolic dysfunction. Therefore, extrapolation across diabetes types should be undertaken cautiously.

### 6.4 Proposed effects on glycaemic and insulin responses

Most evidence comes from adult intervention studies and mechanistic investigations. Evidence in children is limited, and diabetes-specific pediatric evidence is minimal.

Although NNSs are generally considered to have minimal direct metabolic effects, several mechanisms have been proposed through which they may indirectly influence glucose metabolism. Experimental studies investigating primarily sucralose, saccharin, and, to a lesser extent, aspartame have suggested that certain NNSs may interact with intestinal sweet taste receptors and thereby influence incretin secretion and insulin responses (57). However, RCTs conducted predominantly in healthy adults and adults with obesity or diabetes generally report minimal or inconsistent effects on glycaemia and insulinaemia (38, 57).

Because incretin hormones such as glucagon-like peptide-1 (GLP-1) and peptide YY (PYY) contribute to appetite regulation by promoting satiety, delaying gastric emptying, and reducing subsequent energy intake, it has been hypothesized that alterations in incretin secretion induced by certain NNSs could indirectly influence energy balance and, ultimately, body weight (57, 128). Similarly, any NNS-related effects on postprandial glycaemic or insulin responses could theoretically influence hunger, meal timing, or food intake through changes in glucose homeostasis (30, 57). However, RCTs and systematic reviews generally report minimal or inconsistent effects of NNSs on these physiological responses in humans (38, 57). Moreover, there is little evidence that any observed changes translate into clinically meaningful differences in appetite, adiposity, energy intake, or long-term body weight regulation (31, 33).

Similarly, co-consumption of NNSs with carbohydrates has been hypothesized to influence glucose absorption or insulin sensitivity, although available findings remain inconsistent and appear to depend on metabolic phenotype, habitual exposure, and the dietary context in which NNSs are consumed (30). Conditioned insulin responses, including cephalic-phase insulin release, have also been proposed as a potential mechanism (57). This hypothesis is primarily relevant to individuals with preserved endogenous insulin secretion, including healthy individuals and those with T2D, in whom anticipatory insulin release could theoretically influence postprandial glucose homeostasis, appetite regulation, and subsequent energy intake (30, 57). In contrast, because endogenous β-cell function is markedly impaired or absent in most individuals with established T1D, cephalic-phase insulin responses are unlikely to have substantial physiological relevance in this population, where glycemic regulation depends predominantly on exogenous insulin administration (23, 64, 105). Accordingly, although conditioned insulin responses could theoretically influence hunger, meal timing, or energy intake in individuals with preserved endogenous insulin secretion, there is currently no convincing evidence that this mechanism translates into clinically meaningful differences in adiposity or long-term body weight.

In children, these physiological responses may be further modified by developmental changes in insulin sensitivity, pubertal hormonal fluctuations, and diabetes treatment strategies (137). Importantly, any potential physiological effects should be interpreted differently in T1D and T2D because of their distinct insulin physiology and therapeutic management. In T1D, these mechanisms occur within the context of exogenous insulin administration and markedly impaired endogenous β-cell function, whereas in T2D they occur against a background of endogenous insulin resistance and progressive β-cell dysfunction. Consequently, although alterations in glycemic, incretin, or insulin responses could theoretically influence appetite regulation, energy intake, and body weight, current evidence does not demonstrate that these physiological mechanisms translate into clinically meaningful differences in appetite, adiposity, or long-term body weight in children with either T1D or T2D.

## 7 Controversies and limitations

**Figure 5:**
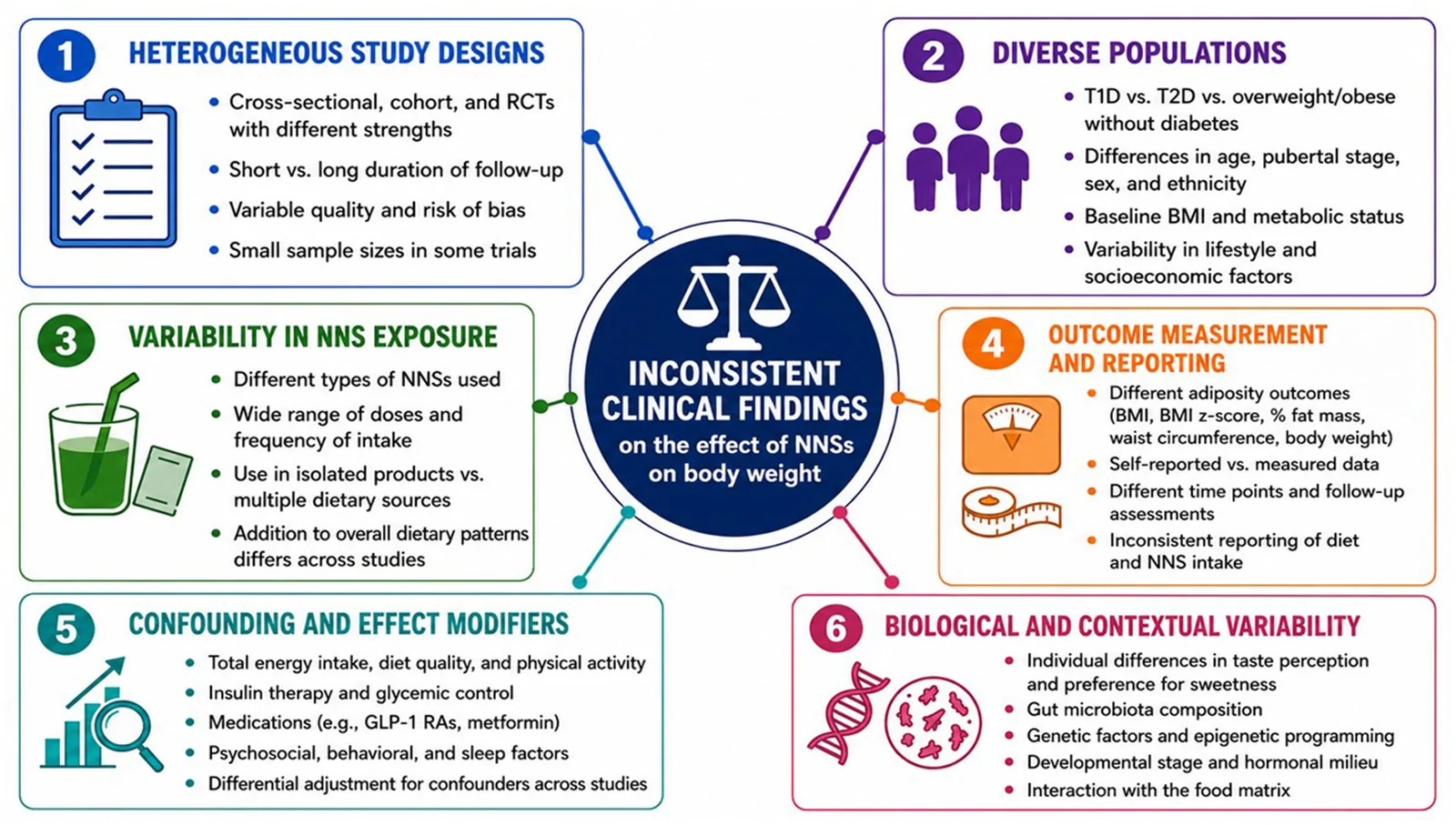
Why are clinical studies inconsistent?

The current body of evidence examining NNSs and body weight outcomes is characterized by substantial methodological, conceptual, and population-specific limitations, which collectively restrict the ability to draw firm causal inferences, particularly in children and adolescents with diabetes (31, 97, 138). The principal methodological and biological factors contributing to the inconsistent findings across the available literature are summarized in Figure 5. Collectively, these sources of heterogeneity complicate the interpretation of the evidence regarding the effects of non-nutritive sweeteners on body weight in children with diabetes.

A remarkable limitation is that pediatric diabetes is frequently considered as a single clinical entity despite the marked differences between type 1 diabetes (T1D) and type 2 diabetes (T2D) in disease pathophysiology, insulin physiology, body weight regulation, and therapeutic objectives. Consequently, extrapolation of findings across diabetes types should be undertaken cautiously, and the available evidence should be interpreted within the specific metabolic and clinical context of each condition.

Another major limitation is the scarcity of studies specifically targeting children with diabetes, despite this being a clinically relevant and metabolically distinct population. Most available evidence is extrapolated from general pediatric cohorts or adult populations, which do not account for the unique physiological characteristics of pediatric diabetes, including exogenous insulin therapy in T1D, insulin resistance and obesity in T2D, and the metabolic variability associated with growth and puberty (136, 137, 139). Moreover, many pediatric investigations either combine participants with T1D and T2D or fail to specify the diabetes type, thereby limiting the interpretation and clinical applicability of their findings. Given the substantial differences between T1D and T2D in body weight regulation, dietary management, and therapeutic goals, extrapolation of findings from one diabetes type to the other may not be appropriate. As a result, external validity remains limited, and conclusions cannot be directly translated into routine clinical practice. The WHO guideline on non-sugar sweeteners also highlights this important evidence gap, emphasizing the lack of robust long-term pediatric disease-specific trials (45).

In addition, existing RCTs are frequently constrained by relatively small sample sizes, short intervention durations, and limited statistical power to detect clinically meaningful differences in body composition and metabolic outcomes. Many studies primarily evaluate intermediate endpoints, such as short-term changes in energy intake or body weight, rather than long-term trajectories of BMI, adiposity, body composition, or cardiometabolic health across critical stages of growth and development (140, 141). Because body weight regulation in children is a slow and multifactorial process influenced by growth, pubertal maturation, dietary behaviors, and treatment regimens, short-term interventions may underestimate or fail to detect delayed or cumulative effects of NNS exposure.

Observational studies, although more reflective of real-world dietary behaviors, are particularly susceptible to confounding and bias. A major concern is reverse causality, whereby children with overweight, obesity, or diabetes who receive dietary counseling may preferentially consume NNS-containing products in an effort to improve glycaemic control or manage body weight (142, 143). Consequently, higher NNS intake may represent a consequence rather than a cause of excess adiposity, resulting in spurious positive associations between NNS consumption and obesity-related outcomes (142, 143). Residual confounding further complicates interpretation because NNS consumption is closely associated with overall dietary patterns, physical activity, sleep duration, socioeconomic status, parental obesity, and other health-related behaviors that cannot always be fully controlled in epidemiological analyses (142, 143). In studies involving children with diabetes, interpretation is further complicated by disease-specific confounding factors, including differences in insulin regimens, total daily insulin dose, carbohydrate counting practices, use of continuous glucose monitoring and insulin pump technologies, frequency of hypoglycaemic episodes, and concomitant pharmacological therapies, all of which may independently influence dietary behavior, energy intake, and body weight regulation.

An additional methodological issue that has received increasing attention concerns the different causal questions addressed by observational studies and RCTs (144, 145). Most observational studies evaluate baseline or prevalent NNS consumption and relate this exposure to subsequent body weight or metabolic outcomes over prolonged follow-up. In contrast, RCTs typically evaluate changes in body weight following experimentally induced replacement of sugar with NNSs. These fundamentally different exposure models are not directly comparable and may partly explain the apparent discrepancies between study designs (144, 145). Importantly, more recent observational investigations adopting longitudinal analytic frameworks that evaluate changes in NNS exposure over time and better emulate the causal contrast assessed in RCTs have reported findings that are generally more consistent with RCT evidence. This suggests that differences between observational and experimental studies may reflect methodological design rather than true biological inconsistency.

Another important limitation concerns the assessment of NNS exposure. Studies vary considerably in the methods used to define and quantify NNS intake, ranging from dietary recalls with limited specificity to food-frequency questionnaires that do not distinguish individual sweeteners or accurately estimate dose. This challenge is further compounded by incomplete food labeling, frequent reformulation of commercial products, and the widespread use of multiple sweeteners within individual food products, making it difficult to isolate the effects of specific compounds. Recent validation studies suggest that exposure misclassification may both attenuate and distort true associations between NNS consumption and health outcomes (146, 147).

Considerable heterogeneity also exists regarding the types, formulations, and doses of NNSs evaluated across studies. Individual sweeteners, including aspartame, sucralose, saccharin, and steviol glycosides, differ substantially in their chemical structure, metabolism, sweetness intensity, gastrointestinal handling, and potential biological effects, yet they are frequently analyzed collectively as a single exposure category. This aggregation may obscure sweetener-specific effects and contribute to inconsistent or conflicting findings across studies. Furthermore, dose-response relationships remain poorly characterized, particularly in children, where exposure may vary considerably according to dietary habits, food availability, product formulation, and marketing practices.

This methodological issue has important implications for the interpretation of the current evidence base. Many randomized controlled trials, observational studies, systematic reviews, and meta-analyses combine chemically distinct NNSs into a single analytical category despite their different physicochemical properties and biological behavior. Such aggregation may increase both clinical and statistical heterogeneity, obscure compound-specific associations, and partly explain the inconsistent or inconclusive findings reported across studies. Moreover, because commercially available foods and beverages frequently contain combinations of multiple sweeteners, potential synergistic or interactive effects remain largely unexplored. Subsequently, conclusions regarding the health effects of “NNSs” should be interpreted cautiously, as pooled estimates may not accurately reflect the biological effects of individual sweeteners. Future evidence syntheses and clinical investigations should therefore evaluate individual NNSs—or clearly defined sweetener combinations—separately whenever feasible.

A further limitation of the current evidence is that many observational studies, RCTs, and meta-analyses evaluate NNSs as a single exposure despite the substantial chemical, metabolic, and physiological differences among individual sweeteners. This approach may obscure compound-specific effects and potentially overlook synergistic or antagonistic interactions between sweeteners that are frequently consumed in combination in commercially available products.

Interpretation of the available evidence is further complicated by the biological and clinical heterogeneity between T1D and T2D. Although dietary management is fundamental in both conditions, body weight regulation in T1D is strongly influenced by exogenous insulin administration and hypoglycaemia management, whereas obesity, insulin resistance, and excess adiposity are primary determinants of body weight in T2D. Consequently, similar dietary interventions, including replacement of sugars with NNS-containing products, may have different physiological consequences and clinical implications according to diabetes type, further emphasizing the need for diabetes-specific investigations.

Differences in study design and outcome measures further limit comparability across investigations. Some studies evaluate BMI z-scores, whereas others assess absolute BMI, waist circumference, body fat percentage, or other anthropometric indices, making direct comparisons challenging. In addition, growth and pubertal maturation are not consistently considered despite their profound influence on body composition and metabolic regulation during childhood and adolescence (148). The absence of standardized outcome definitions and reporting practices substantially limits evidence synthesis and complicates quantitative meta-analyses.

Moreover, considerable scientific uncertainty remains regarding the biological plausibility of NNS-induced effects on body weight regulation. Proposed mechanisms include alterations in appetite regulation, reward processing, gut microbiota composition, glucose homeostasis, and hormonal signaling; however, the consistency and clinical relevance of these pathways in humans remain uncertain. Systematic reviews evaluating microbiome and metabolic outcomes indicate that findings are highly heterogeneous and frequently influenced by study design, baseline dietary habits, and individual metabolic phenotype. Furthermore, much of the mechanistic evidence derives from animal experiments or short-term experimental studies, and its translation to long-term clinical outcomes in children remains unclear. Publication bias and selective reporting may also contribute to the existing literature, as studies reporting statistically significant associations are generally more likely to be published than those reporting neutral findings.

Finally, another important limitation of the current literature is the tendency to frame NNSs as therapeutic interventions rather than dietary substitutions. Many clinical studies implicitly evaluate NNSs as if they were pharmacological agents capable of directly improving metabolic outcomes. However, because the primary purpose of NNSs is to replace sugars, observed benefits should generally be interpreted in the context of reduced sugar intake rather than intrinsic biological activity. Failure to distinguish between these concepts may contribute to overinterpretation of study findings and unrealistic expectations regarding the role of NNSs in obesity and diabetes management.

Overall, these methodological limitations and ongoing controversies indicate that the current evidence remains insufficient to establish causal relationships between NNS consumption and long-term body weight outcomes in children and adolescents with diabetes. They also underscore the importance of interpreting the available literature separately for T1D and T2D, while highlighting the need for well-designed, adequately powered, long-term pediatric studies employing standardized assessment of NNS exposure, rigorous control of confounding variables, and clinically relevant outcome measures stratified by diabetes type. Consistent with the WHO guideline and recent expert consensus statements, a cautious and individualized approach to NNS use in children remains warranted until more robust evidence regarding their long-term safety and metabolic effects becomes available (45). Finally, the factors summarized in Figure 5 highlight why current evidence should be interpreted cautiously and underscore the need for well-designed, adequately powered, long-term randomized controlled trials using standardized assessments of NNS exposure and body weight outcomes in pediatric populations.

## 8 Clinical and dietary implications

The available evidence suggests that NNSs should be considered adjunctive dietary tools rather than stand-alone interventions for body weight management in children with diabetes. Although replacing sugar-containing foods and beverages with NNS-containing alternatives may reduce short-term glycemic load and modestly decrease energy intake, current evidence does not support clinically meaningful long-term reductions in body weight attributable to NNS consumption alone (28, 31, 38, 108). Consequently, NNSs should be incorporated within comprehensive dietary strategies that emphasize healthy eating patterns, regular physical activity, behavioral interventions, and family-centered nutritional education rather than being viewed as primary weight-management therapies (23, 64).

An important conceptual consideration is that NNSs should not be regarded as therapeutic agents. Their primary function is to replace added sugars in foods and beverages, thereby reducing glycemic load and, in some cases, overall energy intake. Accordingly, any observed improvements in glycemic control or body weight should generally be interpreted as indirect consequences of sugar replacement rather than as evidence of intrinsic pharmacological or therapeutic effects of NNSs themselves. This distinction is particularly important in pediatric diabetes, where NNSs should be viewed as adjunctive dietary tools that may support healthy eating patterns rather than as stand-alone therapeutic interventions.

Healthcare professionals should recognize that products labeled as “sugar-free” or “diet” may create a misleading perception that they can be consumed without restriction. Nutritional counseling should therefore emphasize the overall nutritional quality of foods rather than focusing exclusively on sugar or caloric content. Importantly, many commercially available NNS-containing products are ultra-processed; however, the potential health implications are unlikely to result from the degree of processing alone. Rather, concern relates primarily to the nutritional characteristics of many of these products, including high energy density, added fats, refined carbohydrates, sodium, low fiber content, large portion sizes, and high palatability, all of which may promote excess energy intake and adversely affect long-term cardiometabolic health (3, 45). Consequently, dietary counseling should prioritize limiting nutritionally poor ultra-processed foods, while encouraging minimally processed or nutrient-dense alternatives, irrespective of whether they contain NNSs.

Special consideration should also be given to the unique nutritional requirements of children and adolescents. Dietary recommendations should simultaneously support normal growth, pubertal development, and optimal glycemic control while avoiding unnecessarily restrictive eating patterns. Accordingly, decisions regarding the use of NNSs should be individualized and integrated into multidisciplinary diabetes management plans involving healthcare professionals, dietitians, children, and their families (16, 23, 64).

### 8.1 Clinical considerations according to diabetes type

Although several dietary principles regarding NNS use are shared between T1D and T2D, their clinical application should be interpreted within the specific metabolic and therapeutic context of each disease.

In children with T1D, the principal dietary role of NNSs is to reduce the glycaemic impact of dietary sugars while preserving dietary flexibility and facilitating carbohydrate counting within intensive insulin therapy. Because body weight regulation in T1D is strongly influenced by exogenous insulin administration, hypoglycaemia management, and pubertal development, NNSs should primarily be regarded as dietary tools that facilitate glycaemic management rather than as interventions intended to promote weight loss (16, 23, 64, 74).

In children and adolescents with T2D where obesity, excess adiposity, and insulin resistance constitute major therapeutic targets, replacing added sugars with nutritionally appropriate NNS-containing foods and beverages may contribute to lowering total energy intake and supporting body weight management as part of comprehensive lifestyle interventions. Nevertheless, any potential benefit depends largely on the overall quality of the dietary pattern, adherence to broader lifestyle modifications, and sustained behavioral changes rather than on NNS consumption itself (23, 106–108).

Importantly, the available evidence remains substantially stronger for general pediatric populations than for children with diabetes, particularly those with T2D. Most pediatric studies either include mixed diabetes populations or fail to distinguish between diabetes types, thereby limiting the development of disease-specific dietary recommendations. Consequently, current guidance regarding NNS use in pediatric diabetes should be individualized according to diabetes type, metabolic status, growth requirements, and overall dietary quality, while recognizing the limited strength of the available evidence.

Overall, as summarized in Figure 4, the current literature supports a cautious and individualized approach to NNS use in children with diabetes. NNSs may help reduce sugar intake and improve short-term glycemic control when they replace nutritionally poor sugar-containing foods and beverages within an overall healthy dietary pattern; however, they should not be considered primary interventions for body weight management. Instead, optimal long-term outcomes are more likely to be achieved through comprehensive lifestyle strategies that integrate individualized nutritional counseling, healthy dietary patterns, regular physical activity, behavioral support, family engagement, and appropriate pharmacological therapy according to the specific clinical characteristics of children with T1D or T2D (23, 45, 64).

## 9 Future research directions

**Figure 6:**
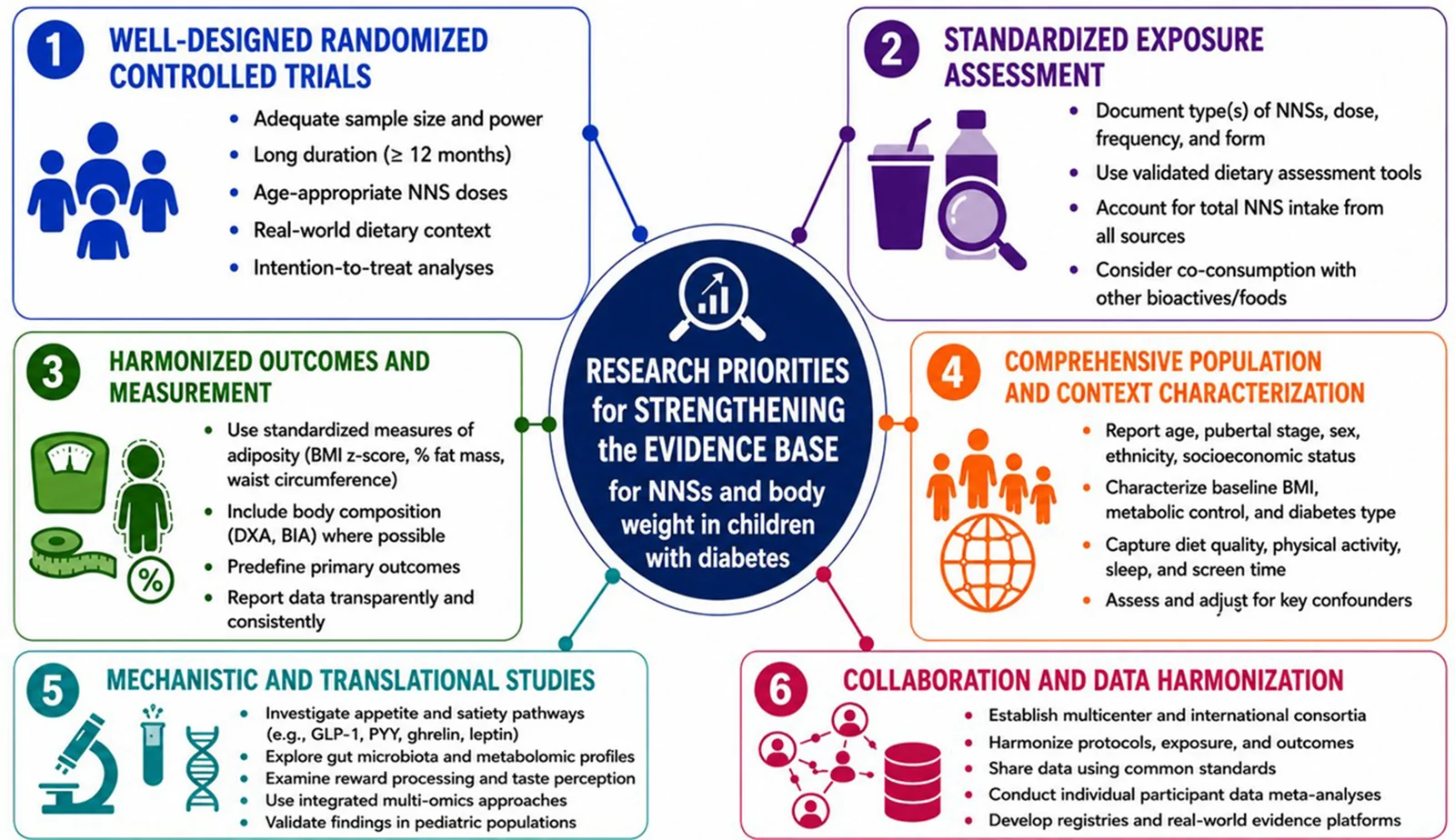
Clinical decision framework for NNS use in pediatric diabetes.

Despite the growing body of literature examining NNSs, important knowledge gaps remain regarding their effects on body weight and metabolic health in children with diabetes. Figure 6 summarizes the key methodological, clinical, and translational priorities that should be addressed in future studies to strengthen the evidence base regarding the effects of non-nutritive sweeteners on body weight in pediatric diabetes.

Future research should address several methodological limitations of the current evidence base while recognizing that T1D and T2D differ substantially in their pathophysiology, insulin physiology, therapeutic objectives, and determinants of body weight regulation. Consequently, future investigations should evaluate these conditions separately whenever possible rather than considering pediatric diabetes as a single clinical entity.

Although long-term RCTs remain the ideal design for evaluating causal effects, their implementation in children with diabetes may be limited by practical, ethical, and feasibility considerations, including concerns regarding prolonged dietary interventions and uncertainty surrounding long-term safety. Consequently, future research should also prioritize high-quality prospective cohort studies that more closely emulate the causal framework of randomized trials. In particular, observational studies should evaluate changes in NNS consumption over time rather than relying solely on baseline exposure, employ repeated dietary assessments, appropriately model sugar replacement, and account for changes in dietary behaviors and potential time-varying confounding. Such designs are likely to provide more clinically relevant estimates of the effects of NNS substitution and complement evidence derived from randomized trials.

Future intervention studies should also employ standardized methodologies for assessing both dietary exposure and clinically relevant outcomes. In addition to body weight and BMI, investigations should evaluate changes in body composition, waist circumference, glycemic control, glycemic variability, insulin requirements, dietary quality, eating behaviors, appetite regulation, total energy intake, physical activity, gut microbiota composition, cardiometabolic risk factors, quality of life, and long-term treatment adherence. Longer follow-up periods extending through critical stages of growth and pubertal development will be essential to determine whether early dietary exposure to NNSs influences long-term metabolic trajectories.

Research priorities are also likely to differ according to diabetes type. In children with T1D, future studies should determine whether NNS consumption influences insulin requirements, glycemic variability, frequency of hypoglycaemic episodes, carbohydrate replacement behaviors, body composition, and long-term weight trajectories within the context of intensive insulin therapy. Because exogenous insulin administration remains the principal determinant of body weight regulation in T1D, future investigations should carefully account for insulin regimen, total daily insulin dose, diabetes duration, and pubertal status when evaluating potential effects of NNSs.

In children and adolescents with T2D, future research should primarily investigate whether replacing added sugars with NNS-containing products contributes to sustained reductions in total energy intake, improvements in body weight management, insulin resistance, glycemic control, and long-term cardiometabolic outcomes when incorporated into comprehensive lifestyle interventions. Given the increasing use of contemporary pharmacological therapies for pediatric T2D, future studies should also evaluate potential interactions between NNS consumption and glucose-lowering medications that independently influence appetite regulation, energy intake, and body weight.

Additional research is required to clarify the biological mechanisms through which NNSs may influence body weight regulation in pediatric diabetes. Future mechanistic studies should integrate metabolic phenotyping, hormonal profiling, continuous glucose monitoring, microbiome analyses, and behavioral assessments to better understand how appetite regulation, reward pathways, energy compensation, gut microbiota alterations, and glycemic responses interact under different metabolic conditions. Such investigations may help explain the considerable inter-individual variability observed across clinical studies.

Methodological improvements should also include more accurate assessment of NNS exposure. Future epidemiological studies should distinguish between individual sweeteners rather than treating NNSs as a single homogeneous category, quantify habitual intake using validated dietary assessment tools and objective biomarkers whenever feasible, and account for overall dietary patterns, food processing level, socioeconomic factors, physical activity, sleep, and other potential confounding variables. Standardized reporting of NNS dose, duration of exposure, and food sources would substantially improve comparability across studies. Similarly, future systematic reviews and meta-analyses should avoid pooling chemically distinct NNSs whenever sufficient data are available, as compound-specific analyses are likely to provide more biologically plausible and clinically meaningful conclusions while reducing unnecessary clinical and statistical heterogeneity.

Future evidence synthesis should also distinguish observational studies according to their underlying analytical framework. Studies evaluating prevalent NNS consumption at baseline address a fundamentally different research question from those assessing changes in NNS intake or explicit substitution of sugars with NNSs. Consequently, systematic reviews and meta-analyses should consider these study designs separately whenever possible rather than pooling them indiscriminately. Such an approach may reduce heterogeneity, improve causal interpretation, and facilitate more meaningful comparison with randomized controlled trials.

Beyond improving methodological quality, future research should also address an important conceptual issue that has received relatively little attention. Specifically, studies should distinguish the effects attributable to sugar replacement from any potential compound-specific biological effects of individual NNSs. Because NNSs are food additives intended primarily to replace added sugars rather than therapeutic agents, future investigations should avoid implicitly assuming that improvements in glycemic control or body weight arise from intrinsic pharmacological effects of NNSs themselves. Instead, intervention studies should determine whether observed benefits primarily reflect reduced sugar consumption or specific physiological actions of individual sweeteners. Clarifying this distinction will improve interpretation of the existing evidence, facilitate more meaningful evidence synthesis, and provide a stronger scientific foundation for evidence-based dietary recommendations and future clinical guidelines.

Finally, future research should move beyond determining whether NNSs simply influence body weight and instead identify which pediatric populations are most likely to benefit, under which dietary circumstances, and whether these effects differ between T1D and T2D. Such evidence will be essential for developing evidence-based, diabetes-specific dietary recommendations regarding the appropriate use of NNSs in children and adolescents. Until such evidence becomes available, clinical recommendations should remain individualized and integrated within comprehensive lifestyle interventions that promote healthy dietary patterns, optimal glycemic control, and long-term cardiometabolic health. Collectively, the research priorities summarized in Figure 6 provide a roadmap for future investigations and may facilitate the development of more evidence-based dietary recommendations regarding non-nutritive sweetener use in children with diabetes.

## 10 Conclusions

Current evidence indicates that replacing added sugars with NNSs may provide short-term dietary benefits for children with diabetes by reducing sugar intake and glycemic load. However, available evidence does not support a clinically meaningful long-term effect of NNS consumption on body weight. Interpretation of the existing literature is complicated by substantial methodological heterogeneity, limited diabetes-specific evidence, short follow-up periods, and the potential influence of reverse causality and residual confounding in observational studies.

Importantly, the available evidence should not be interpreted uniformly across pediatric diabetes. Children with T1D and T2D differ fundamentally in their pathophysiology, insulin physiology, determinants of body weight regulation, and therapeutic goals. In T1D, the principal value of NNSs lies in reducing the glycemic impact of dietary sugars while facilitating carbohydrate counting and preserving dietary flexibility within intensive insulin therapy, whereas body weight regulation remains largely influenced by exogenous insulin administration and hypoglycaemia management. In contrast, in pediatric T2D, where obesity and insulin resistance are central disease characteristics, replacing added sugars with NNS-containing products may contribute to reducing energy intake and supporting body weight management as part of comprehensive lifestyle interventions. Nevertheless, direct evidence in children with T2D remains extremely limited.

Accordingly, NNSs should be regarded as adjunctive dietary tools rather than therapeutic agents or primary weight-management interventions. Their principal role is to replace added sugars in foods and beverages, thereby reducing glycemic load and, under appropriate dietary circumstances, energy intake, rather than exerting intrinsic pharmacological or therapeutic effects. Consequently, any improvements in glycemic control or body weight should generally be interpreted as indirect consequences of sugar replacement within an overall healthy dietary pattern rather than as direct effects of NNSs themselves. Their use should therefore always be integrated within individualized nutritional counseling that emphasizes healthy dietary patterns, regular physical activity, behavioral support, family engagement, and appropriate medical therapy according to diabetes type.

Future well-designed, adequately powered randomized controlled trials should evaluate children with T1D and T2D separately, using standardized assessment of NNS exposure and clinically relevant outcomes, including body composition, glycemic control, insulin requirements, cardiometabolic health, and long-term safety. Such evidence will be essential for developing evidence-based, diabetes-specific dietary recommendations regarding the appropriate use of NNSs in children and adolescents.
