# Supplementary material for: Non-nutritive sweeteners and body weight in children with type 1 and type 2 diabetes: a critical narrative review of current evidence, methodological challenges, biological mechanisms, and clinical implications
Source: Front Nutr. 2026 Aug 27;13:1892085. doi: 10.3389/fnut.2026.1892085 (PMC13559788; doi:10.3389/fnut.2026.1892085)
Supplement: Supplementary file 1 [file Table_1.docx]

Supplementary Table S1. Literature search strategies used for each electronic database.

| **Database** | **Search strategy** |
| --- | --- |
| **PubMed** | ("non-nutritive sweetener*" OR "artificial sweetener*" OR "low-calorie sweetener*" OR "high-intensity sweetener*" OR aspartame OR sucralose OR saccharin OR acesulfame-K OR acesulfame potassium OR cyclamate OR neotame OR advantame OR stevia OR "steviol glycoside*" OR "monk fruit" OR mogroside*) AND ("type 1 diabetes" OR T1D OR "type 2 diabetes" OR T2D OR diabetes) AND (child* OR adolescen* OR pediatric OR paediatric) AND ("body weight" OR obesity OR overweight OR adiposity OR BMI OR "weight gain" OR "weight management") |
| **Scopus** | Same concepts adapted to Scopus syntax |
| **Web of Science** | Same concepts adapted to WoS syntax |
| **Google Scholar** | ("non-nutritive sweeteners" diabetes children body weight) together with combinations of the names of individual sweeteners and relevant outcome terms |
